# BMP2-modified injectable hydrogel for osteogenic differentiation of human periodontal ligament stem cells

**DOI:** 10.1038/s41598-017-06911-8

**Published:** 2017-07-26

**Authors:** Seung Hun Park, Jin Seon Kwon, Byeong Sung Lee, Ji Hoon Park, Bo Keun Lee, Jeong-Ho Yun, Bun Yeoul Lee, Jae Ho Kim, Byoung Hyun Min, Tae Hyeon Yoo, Moon Suk Kim

**Affiliations:** 1 Department of Molecular Science and Technology, Ajou University, Suwon, 443-749 Korea; 2 Department of Periodontology, School of Dentistry and Institute of Oral Bioscience, Chonbuk National University, Jeonju, 561-712 Korea

## Abstract

This is the first report on the development of a covalently bone morphogenetic protein-2 (BMP2)-immobilized hydrogel that is suitable for osteogenic differentiation of human periodontal ligament stem cells (hPLSCs). *O*-propargyl-tyrosine (OpgY) was site-specifically incorporated into BMP2 to prepare BMP2-OpgY with an alkyne group. The engineered BMP2-OpgY exhibited osteogenic characteristics after *in vitro* osteogenic differentiation of hPLSCs, indicating the osteogenic ability of BMP2-OpgY. A methoxy polyethylene glycol-(polycaprolactone-(N_3_)) block copolymer (MC-N_3_) was prepared as an injectable *in situ*-forming hydrogel. BMP2 covalently immobilized on an MC hydrogel (MC-BMP2) was prepared quantitatively by a simple biorthogonal reaction between alkyne groups on BMP2-OpgY and azide groups on MC-N_3_ via a Cu(I)-catalyzed click reaction. The hPLSCs-loaded MC-BMP2 formed a hydrogel almost immediately upon injection into animals. *In vivo* osteogenic differentiation of hPLSCs in the MC-BMP2 formulation was confirmed by histological staining and gene expression analyses. Histological staining of hPLSC-loaded MC-BMP2 implants showed evidence of mineralized calcium deposits, whereas hPLSC-loaded MC-Cl or BMP2-OpgY mixed with MC-Cl, implants showed no mineral deposits. Additionally, MC-BMP2 induced higher levels of osteogenic gene expression in hPLSCs than in other groups. In conclusion, BMP2-OpgY covalently immobilized on MC-BMP2 induced osteogenic differentiation of hPLSCs as a noninvasive method for bone tissue engineering.

## Introduction

Bone morphogenetic proteins (BMPs) are some of the most commonly used growth factors in orthopedic applications^1, 2^. BMPs have the U.S. Food and Drug Administration approval for clinical treatments for mainly spine fusion including long bone fracture fixation, ankle fusion, and vertebral fracture repair^3^. Among the BMPs, BMP2 can cause more bone overgrowth than other BMPs^4^. BMP2 can regenerate bone through osteogenic differentiation of osteoblast progenitors or stem cells^5^.

Clinical administration of BMP2 has sometimes required focal repeated exposures to BMP2 at supra-physiological doses to achieve the biologic activity needed for remedial effects because of its comparatively short half-life *in vivo* ^6^. This repeated administration at supra-physiological doses is costly and may also lead to unfavorable side effects, including excessive remedial effects and adverse immune responses^7^.

A number of attempts to develop alternative drug delivery formulations have been made to minimize unfavorable side effects and to optimize the remedial effects of BMP2^8–10^. Generally, BMP2 can be loaded in drug delivery formulations by simple mixing (physical adsorption via non-covalent bonding). As an example, a BMP2 protein that includes a material-binding motif and heparin-grafted materials for interaction with BMP2 has been developed. The approach allows timed release through intermolecular interactions between the materials and BMP2^11, 12^.

However, BMP2 does not remain in a BMP2-loaded carrier after a certain period of time^13^. The content and conformation of BMP2 cannot be precisely controlled after physical adsorption. In certain cases, physical adsorption may not provide BMP2 efficacy and stability for the needed period of time^14^.

It is conceivable that covalent immobilization of BMP2 on a drug carrier could allow sufficient and sustained administration of BMP2 to maintain physiological levels and prolong its half-life^15, 16^. However, this method usually requires a functional group to anchor the BMP2 directly to the drug carrier material. Thus, a carrier material and BMP2 with specific functional groups are required.

Usually, the nucleophiles of natural amino acids such as amine or sulfohydryl have been used to conjugate BMP2^17^. However, there is more than one reactive group in BMP2, and this method might suffer from poor site-specificity and heterogeneous products. In addition, non-specific chemical modifications can result in inactivation of proteins or their incorrect orientation^18–21^.

To address these issues of previous methods, we incorporated abiological amino acids into the target BMP2 protein. Several laboratories have developed methods to produce recombinant proteins with unnatural amino acids^22^, and we focused on the orthogonal tRNA_CUA_ (MjtRNA_CUA_) and tyrosyl-tRNA synthetase (MjTyrRS) pairs from *Methsanococcus jannaschii* ^23^. These pairs have been engineered to incorporate various unnatural amino acids into the TAG stop codon^24–27^, and a number of successful applications of the system have been reported^21, 28–31^. Among the amino acid analogues, we chose *O*-propargyl-tyrosine (OpgY) for incorporation into the target BMP2 (BMP2-OpgY). Thus, the first aim of this work was to design and prepare BMP2-OpgY engineered with an alkyne group and then to examine its ability to induce osteogenic differentiation of human periodontal ligament stem cells (hPLSCs).

In recent years, several click reactions have been used for rapid chemical reactions at physiologically relevant conditions^32^. Among the click reaction, the Cu(I)-catalyzed reaction between alkyne and azide groups can cause a rapid bioorthogonal reaction in aqueous media as well as under physiological conditions^33^. Thus, we hypothesized that the BMP2-OpgY prepared with an alkyne group could be used in a click reaction to achieve a bioorthogonal reaction.

Injectable *in situ*-forming hydrogels appear to be a promising drug carrier. These hydrogels can incorporate various biologic components such as cells, growth factors, and drugs, simply by mixing at room temperature (RT)^34^. Furthermore, injectable hydrogels can be administrated by a simple injection, resulting in only minor discomfort for the patient.

Recently, we reported a methoxy polyethylene glycol-polycaprolactone block copolymer (MPEG-PCL [MC]) as an injectable *in situ*-forming hydrogel for the delivery of several biologic factors^35–39^. Because aqueous solutions of MC exhibited the solution to gelation transition at body temperatures, subcutaneous injection of an MC solution into an animal resulted in the rapid formation of a hydrogel depot.

If we added BMP2 into the MC solution, the MC hydrogel loaded with BMP2 via physical adsorption did not remain in the MC hydrogel after a certain treatment time^40, 41^. We hypothesized that an azide group on an MC carrier could covalently immobilize BMP2-OpgY on that carrier. Thus, the second aim of this work was to design and prepare an azide-modified MC hydrogel and then to exploit the click reaction between the alkyne group of BMP2-OpgY and azide group of MC in order to create a BMP2-modified MC (MC-BMP2) that was capable of rapid hydrogel formation.

Human teeth can be easily obtained during dental repair or surgery, and teeth provide human periodontal ligament stem cells (hPLSCs) in large quantities. hPLSCs contain a stromal population of cells that are self-renewing and multipotent; in addition, they have a high proliferation rate and can be cryopreserved for long periods, making them attractive for clinical studies. More importantly, hPLSCs are capable of differentiation into an osteogenic lineage, as well as differentiation into other cells, such as those in odontoblastic, neurogenic, and adipocytic lineages^42^. We chose hPLSCs as a cell source in this work and examined an injectable hPLSC-incorporated MC-BMP2 formulation *in vivo*.

**Figure 1:**
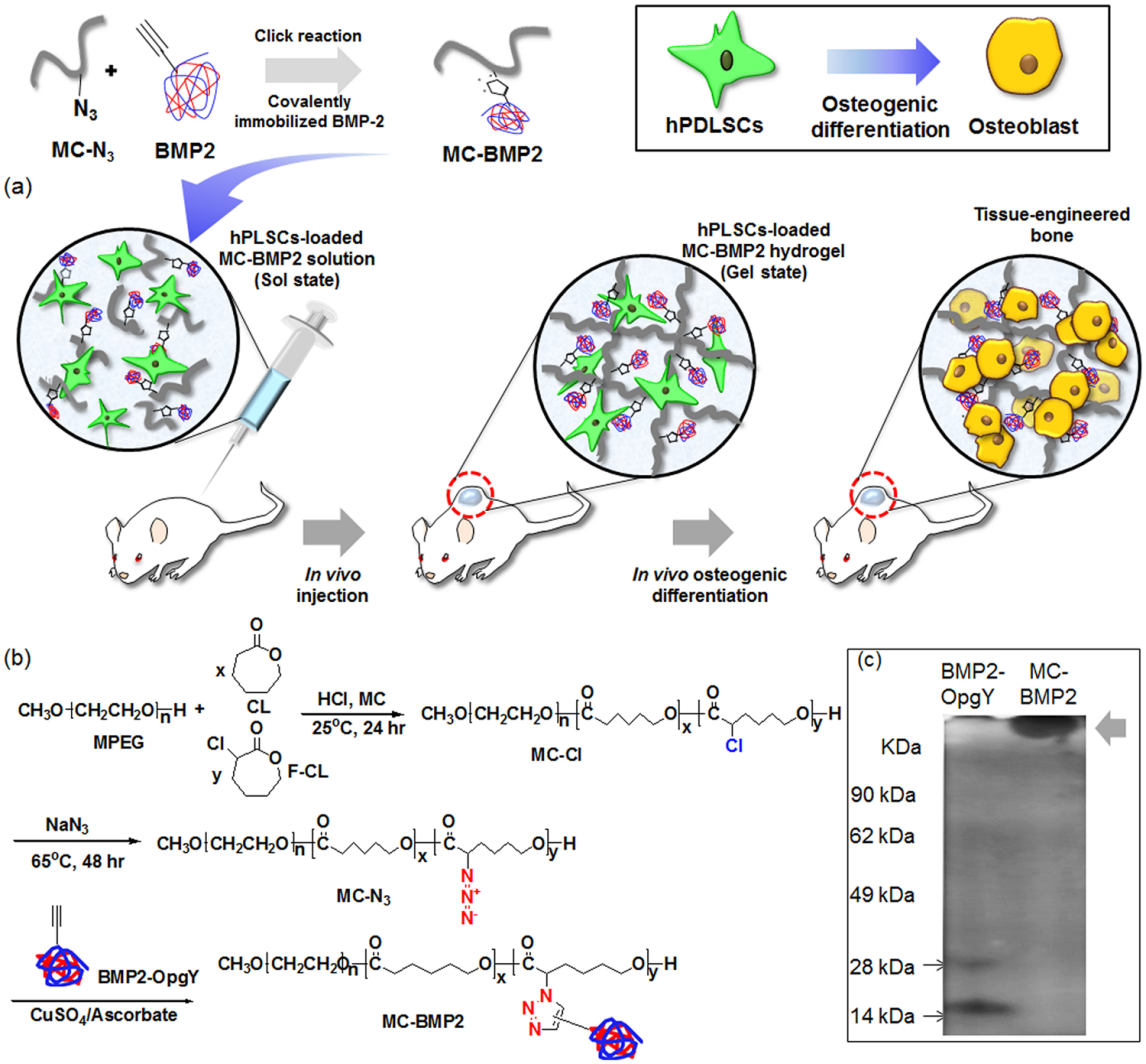
(**a**) Schematic of osteogenic differentiation of human periodontal ligament stem cells in an *in vivo*-formed MC-BMP2 hydrogel, (**b**) synthesis of MC-Cl, MC-N_3_, and MC-BMP2 and (**c**) sodium dodecyl sulfate-polyacrylamide gel electrophoresis of BMP2-OpgY and MC-BMP2. Sulfate-polyacrylamide gel was cropped and full-length gel included in Supplementary Fig. S6a.

This is the first report of the development of an injectable MC-BMP2 formulation that is suitable for osteogenic differentiation of hPLSCs (Fig. 1a). Therefore, the objectives of the current study were (1) to prepare BMP2-OpgY with an alkyne group and to examine the osteogenic differentiation of hPLSCs induced by BMP2-OpgY, (2) to evaluate whether covalently immobilized MC-BMP2 formulated by a quick click reaction can be injected to produce a hydrogel (Fig. 1b), and (3) to determine whether the injectable MC-BMP2 can induce synergistic osteogenic differentiation of hPLSCs that is greater than that of a physically BMP2-loaded MC hydrogel (MC-Cl [+BMP2-OpgY]). Results will indicate the potential for (pre)clinical orthopedic administration of BMP2-modified MC.

## Results and Discussion

### Preparation of BMP2-OpgY

**Figure 2:**
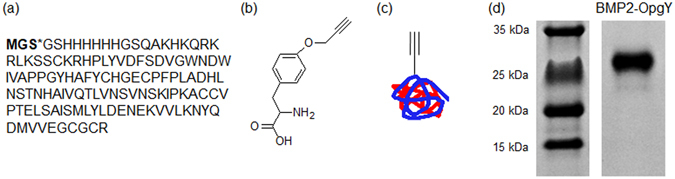
(**a**) Amino acid sequence of BMP2-OpgY, (**b**) chemical structure of *O*-propargyl-tyrosine (OpgY), (**c**) schematic image of BMP2-OpgY, and (**d**) sodium dodecyl sulfate-polyacrylamide gel electrophoresis of BMP2-OpgY. Sulfate-polyacrylamide gel was cropped and full-length gel included in Supplementary Fig. S6b.

To prepare BMP2-OpgY with an alkyne group (Fig. 2), a TAG stop codon for OpgY incorporation was inserted at the fourth position of the recombinant BMP2 because a genetic modification at the *C*-terminus of BMP2 was reported to abolish its biological activities^22^ and the *N*-terminal region of BMP2 is not involved in interactions with its receptors. Using the engineered MjTyrRS - MjtRNA_CUA_ described in the experimental section, OpgY was incorporated into the TAG position. The recombinant BMP2-OpgY proteins expressed as inclusion bodies were refolded, and then their dimeric forms (29.4 kDa) were purified via a heparin column (Fig. 2d).

### Preparation and characterization of hPLSCs

hPLSCs, isolated from a young woman, proliferated rapidly in culture medium and were expanded up to five passages. The expression of surface antigens by hPLSCs was evaluated using flow cytometry. Passage five hPLSCs were positive for the mesenchymal stem cell markers CD90 (>99.9%) and CD166 (>99.8%) and negative for the hematopoietic stem cell marker CD34 (<0.01%) (Supplementary Fig. 1). hPLSCs maintained stem cell characteristics throughout passages one to five. These results indicated that hPLSCs could be used as a stem cell source for osteogenic differentiation in the subsequent experiment.

### *In vitro* osteogenic differentiation of hPLSCs by BMP2-OpgY

**Figure 3:**
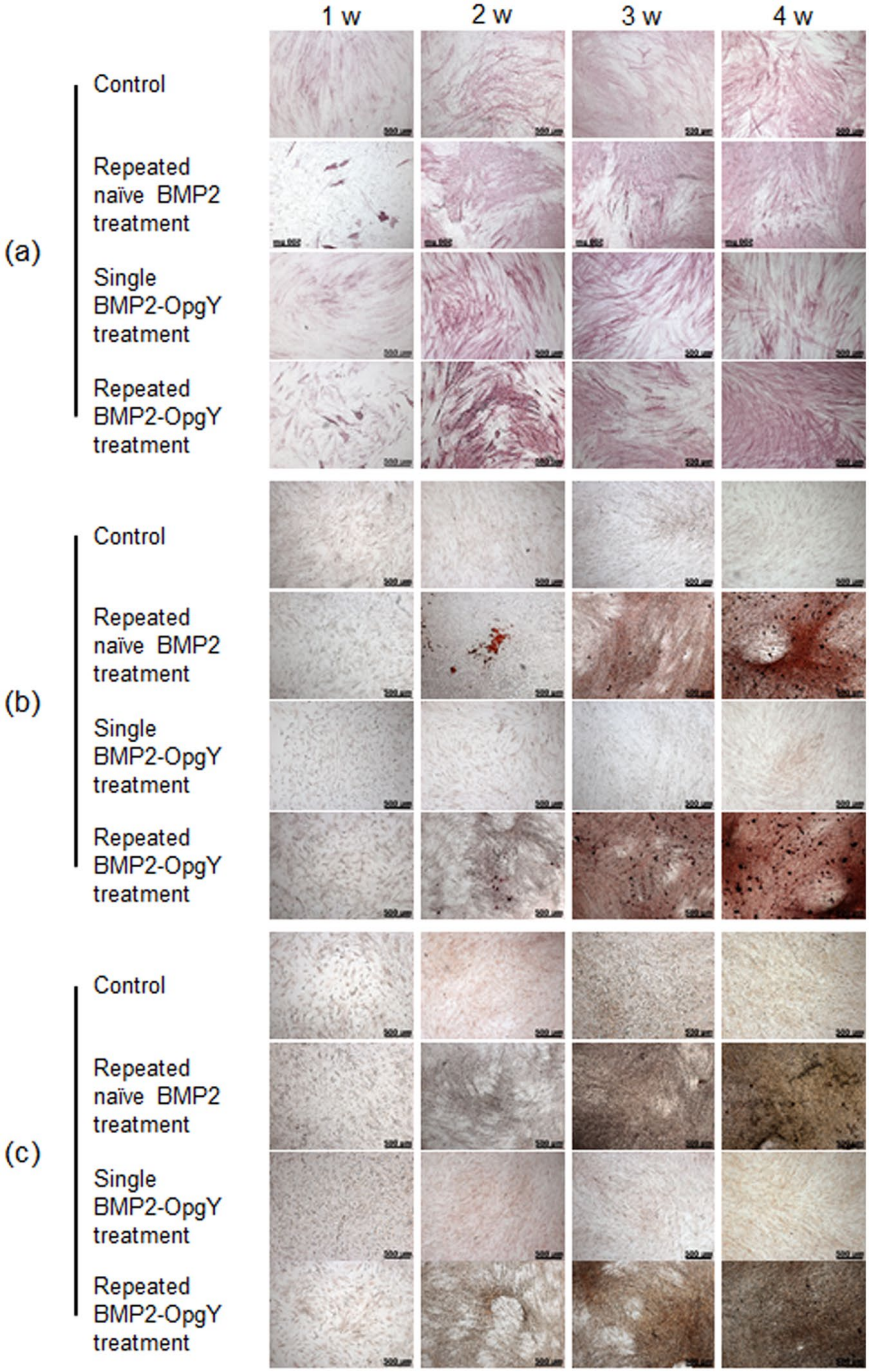
Staining with (**a**) alkaline phosphatase (ALP), (**b**) Alizarin Red (ARS), and (**c**) von Kossa (VK) of human periodontal ligament stem cells grown in culture medium (control), treated with a single exposure to BMP2-OpgY, and treated with repeated exposure to naïve BMP2 or BMP2-OpgY (magnification 50×, scale bars represent 500 μm).

We examined osteogenic differentiation of hPLSCs by the naïve BMP2 and engineered BMP2-OpgY. The hPLSCs were cultured for 4 weeks in culture medium (control), treated with osteogenic medium with BMP2-OpgY for 3 days and then cultured in culture medium (single BMP2-OpgY treatment), or treated with osteogenic medium with naïve BMP2 or BMP2-OpgY changed every 3 days (repeated naïve BMP2 or BMP2-OpgY treatment). Osteogenic characteristics induced by naïve BMP2 or BMP2-OpgY were identified by alkaline phosphatase (ALP), Alizarin red S (ARS) and von Kossa (VK) staining (Fig. 3).

hPLSCs in control and single BMP2-OpgY-treated hPLSCs revealed few or no osteogenic characteristics even after 4 week of culture. This result indicates that hPLSCs could not differentiate without naïve BMP2 or BMP2-OpgY and also did not differentiate with single BMP2-OpgY treatment.

In contrast, the hPLSCs treated with repeated exposure to naïve BMP2 or BMP2-OpgY exhibited osteogenic characteristics by ALP, ARS and VK staining. At week 1, the hPLSCs with repeated naïve BMP2 or BMP2-OpgY treatment exhibited few osteogenic characteristics, similar to the control and single BMP2-OpgY-treated hPLSCs. The hPLSCs exhibited a faint pink color at 2 weeks that became deep pink at 3 and 4 weeks of culture with ALP staining. With ARS staining, the formation of mineralized nodules was observed as a brown color at 2 weeks that darkened and became more widely distributed at 3 and 4 weeks. VK staining produced a light gray color at 2 weeks that became dark gray at 3 and 4 weeks, indicating the formation of calcium deposits. Quantitative analysis of ARS and VK showed a similar osteogenic characteristic in the hPLSCs treated with repeated exposure to naïve BMP2 or BMP2-OpgY (Supplementary Fig. S2).

These data confirm that the engineered BMP2-OpgY prepared in this study could induce osteogenic differentiation of hPLSCs, but that osteogenic differentiation of hPLSCs required treatment with engineered BMP2-OpgY for at least 3 weeks.

### Preparation of the injectable MPEG-b-(PCL-ran-PfCL-N_3_) diblock copolymer (MC-N_3_) hydrogel

Figure 1b shows the scheme for preparing MPEG-b-(PCL-ran-PfCL-Cl) diblock copolymer (MC-Cl) and MC-N_3_. MC-Cl was prepared by ring-opening polymerization of the monomers CL and fCL using the terminal alcohol of MPEG as the initiator. The colorless MC-Cl diblock copolymers were obtained with a 90% yield. The carbons of carbonyl in PCL and PfCL-Cl segments were observed at *δ* = 172.7 and 169.6 ppm at ^13^C-NMR peaks (Supplementary Fig. S3). The ratios of PCL and PfCL-Cl segments in copolymers were determined by the carbon integration ratios of carbonyl in PCL and PfCL-Cl segments using ^13^C-NMR spectra, which agreed well with the expected 91:9 values.

MC-N_3_ was prepared by reaction of sodium azide with MC-Cl, resulting in an 89% yield. MC-N_3_ diblock copolymers also exhibited characteristic peaks in ^1^H- and ^13^C-NMR. The chloro-bonding carbon in MC-Cl segments completely disappeared at *δ* = 169.6 in ^13^C-NMR peaks, and azide bonding in MC-N_3_ segments was observed at *δ* = 62.0 pm in ^13^C-NMR peaks (Supplementary Fig. S3). The polydispersity indexes of MC-Cl and MC-N_3_ after reaction were maintained at about 1.3. This result indicated that the pendant azide group was stoichiometrically introduced into the MC diblock copolymer, creating an active site for a click reaction of BMP2-OpgY with the alkyne group.

### Preparation and characterization of the injectable MPEG-b-(PCL-ran-PfCL-BMP2) diblock copolymer (MC-BMP2) hydrogel

MC-N_3_ and BMP2-OpgY were individually prepared as described in the experimental section. MC-BMP2 was prepared by a simple biorthogonal reaction between the alkyne group on BMP2-OpgY and the azide group on MC-N_3_ via a Cu(I)-catalyzed click reaction.

In the sodium dodecyl sulfate-polyacrylamide gel electrophoresis (SDS-PAGE) analysis of BMP2-OpgY and MC-BMP2, the sizes of BMP2-OpgY in dimeric and monomeric form were 29.4 and 14.7 kDa, respectively; because the sample buffer for SDS-PAGE included a reducing reagent, the both forms were observed. MC-BMP2 was large-sized (Fig. 1c), suggesting that the MC chain of BMP2 was modified. Furthermore, the engineered MC-BMP2 showed no unreacted BMP2-OpgY, indicating a quantitative biorthogonal reaction between the alkyne group on BMP2-OpgY and the azide group of MC-N_3_.

### Characterization of MC-BMP2 solutions

The MC-Cl solution alone, BMP2-OpgY physically loaded onto MC-Cl and MC-BMP2 chemically modified by BMP2-OpgY as injectable *in vivo*-forming hydrogels were prepared to examine the phase-transition. An MC-Cl solution was physically mixed with BMP2-OpgY [MC-Cl (+BMP2-OpgY)], because MC-Cl cannot react with the alkyne of BMP2-OpgY.

**Figure 4:**
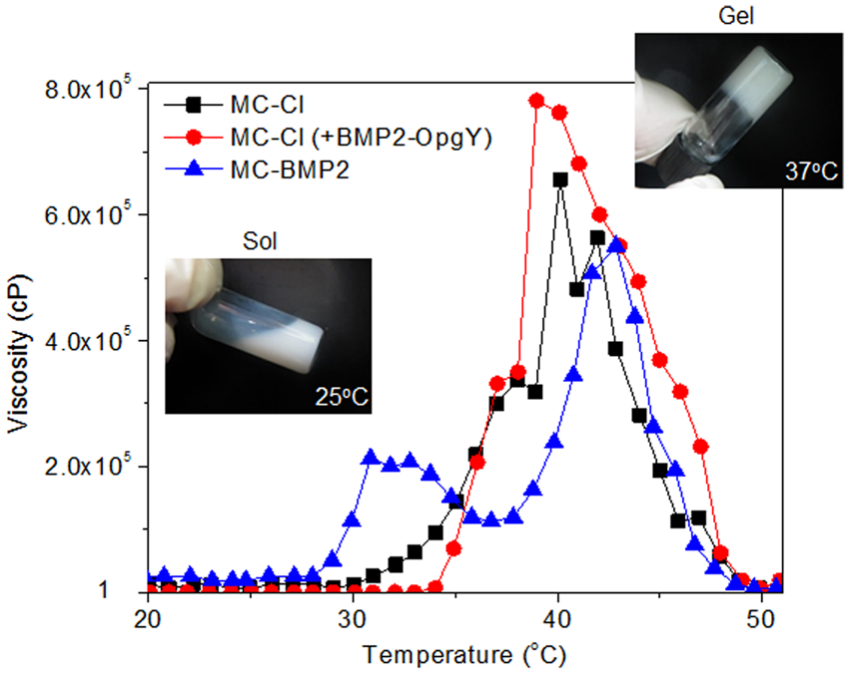
Viscosity vs. temperature curves of MC-Cl, MC-Cl (+BMP2-OpgY), and MC-BMP2.

The prepared MC-Cl, MC-Cl (+BMP2-OpgY), and MC-BMP2 solutions exhibited a translucent emulsion-like solution and flowed through tilting at RT as shown in Fig. 4. All solutions became a hydrogel at 37 °C. The typical transition time in the tilting experiment with the MC-Cl, MC-Cl (+BMP2-OpgY), and MC-BMP2 solutions was about 30 s at 37 °C.

The viscosities of MC-Cl, MC-Cl (+BMP2-OpgY), and MC-BMP2 were measured as a temperature function from 20 °C to 52 °C (Fig. 4). At ambient temperatures up to 28 °C, the viscosities of all hydrogel solutions were 1 cP, reflecting their emulsion-sol state. The viscosities of MC-Cl, MC-Cl (+BMP2-OpgY), and MC-BMP2 began to increase at 30, 34 and 28 °C, respectively, indicating that the onset temperatures for gelation of the hydrogels were affected by BMP2.

The viscosities at body temperature (37 °C) were 3.31 × 10^5^ cP for the MC-Cl hydrogel, 3.00 × 10^5^ cP for the MC-Cl (+BMP2-OpgY) hydrogel, and 1.15 × 10^5^ cP for the MC-BMP2 hydrogel. There was little change in the viscosity of MC-Cl because BMP2-OpgY as added to the MC-Cl solution. However, the viscosity of MC-BMP2 was slightly lower than those of MC-Cl and MC-Cl (+BMP2-OpgY) at 37 °C. Despite its slightly low viscosity, the MC-BMP2 solution became a hydrogel at body temperature.

Meanwhile, the viscosity of the MC-BMP2 slightly decreased above 32 °C and then it increased again above 38 °C. We conjectured that this phenomenon of MC-BMP2 was associated with the pre-aggregated gel formation at 28–36 °C and then more tightly aggregated gel formation above 38 °C. This indicated that MC-BMP2 chemically modified with BMP2-OpgY may have affected the formation of a structured network and, therefore, the aggregation effect of PCL hydrophobic blocks in MC. Collectively, the MC-BMP2 solution was used as an injectable hydrogel in the following experiment.

### *In vitro* release

The *in vitro* release of naïve BMP2 or BMP2-OpgY from the MC-Cl (+naïve BMP2) and MC-BMP2 hydrogels was examined at 37 °C for 21 days (Supplementary Fig. S4). The cumulative release of naïve BMP2 from the MC-Cl (+naïve BMP2) hydrogel was 14% at 1 day and approximately 45% at 21 days. This result indicated that naïve BMP2 loaded via physical adsorption was consistently released from MC-Cl (+naïve BMP2). Meanwhile, as expected no BMP2-OpgY was observed MC-BMP2 hydrogel even at 21 days. Thus, we confirmed that BMP2-OpgY was completely covalently immobilized BMP2-OpgY on MC.

### *In vitro* proliferation of hPLSCs

**Figure 5:**
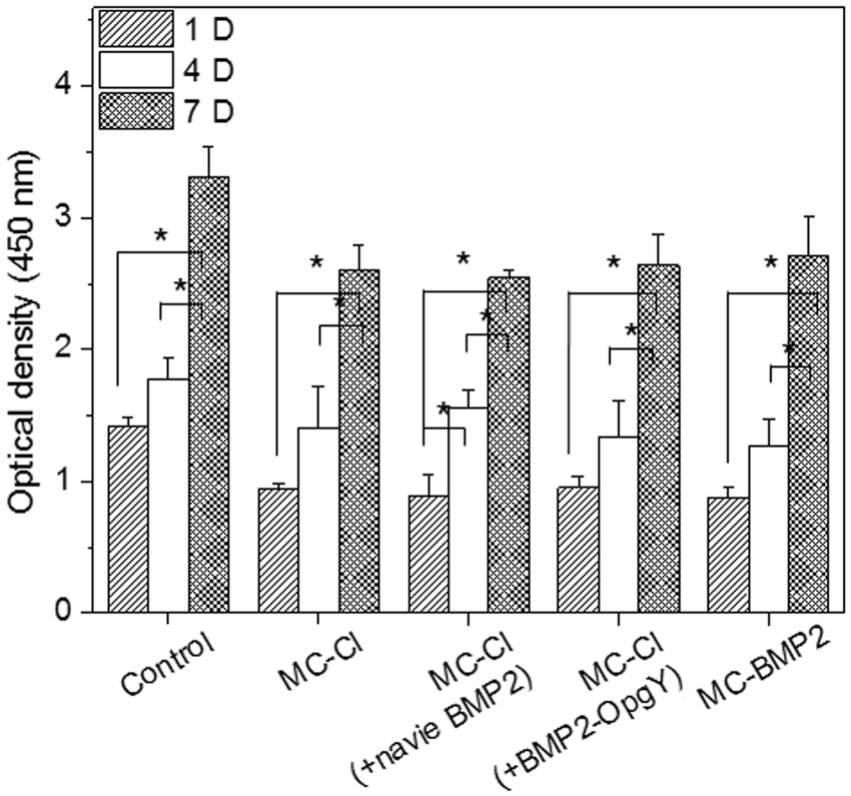
Viability of hPLSCs on culture plates or MC-Cl, MC-Cl (+naïve BMP2), MC-Cl (+BMP2-OpgY) and MC-BMP2 hydrogels measured with the WST-1 assay. Statistical analysis was per-formed using one-way ANOVA with Bonferroni’s multiple comparison correction (*p < 0.01).

hPLSCs attachment and growth were examined to evaluate the biocompatibility of the MC-Cl, MC-Cl (+naïve BMP2), MC-Cl (+BMP2-OpgY), and MC-BMP2 hydrogels over a 7-day incubation period (Fig. 5). hPLSCs as control were monitored in culture plates for comparison. The hPLSCs gradually proliferated in all hydrogels as well as the culture plate. The viability of hPLSCs in MC-Cl, MC-Cl (naïve BMP2), MC-Cl (+BMP2-OpgY), and MC-BMP2 hydrogels was about 70% compare to that in the culture plate. This indicates that all hydrogel exhibited suitable biocompatibility for hPLSCs. Thus the MC-Cl, MC-Cl (+BMP2-OpgY), and MC-BMP2 formulations were used in the following *in vivo* experiment.

### *In vivo* implantation

To examine the osteogenic differentiation of hPLSCs in injectable hydrogels *in vivo*, MC-Cl, MC-Cl (+BMP2-OpgY), and MC-BMP2 loaded with hPLSCs and in solution were subcutaneously injected into nude mice. Immediately after injection, the hPLSC-loaded MC-Cl, MC-Cl (+BMP2-OpgY), and MC-BMP2 solutions formed hydrogels.

The hPLSC-loaded MC-Cl, MC-Cl (+BMP2-OpgY), and MC-BMP2 hydrogels removed at 2, 4, or 8 weeks after injection maintained their shapes through the 8-week implantation period (Supplementary Fig. S5). The removed hydrogels exhibited thin fibrous capsules with fibroblasts, with blood vessels around the hydrogels after 2–8 weeks. Field-emission scanning electron microscopy (FE-SEM) images of the removed hydrogels showed a porous inner structure with interconnected channels (Supplementary Fig. S5). All hydrogel implants maintained their three-dimensional interconnected pore structures through the 8-week implantation period.

**Figure 6:**
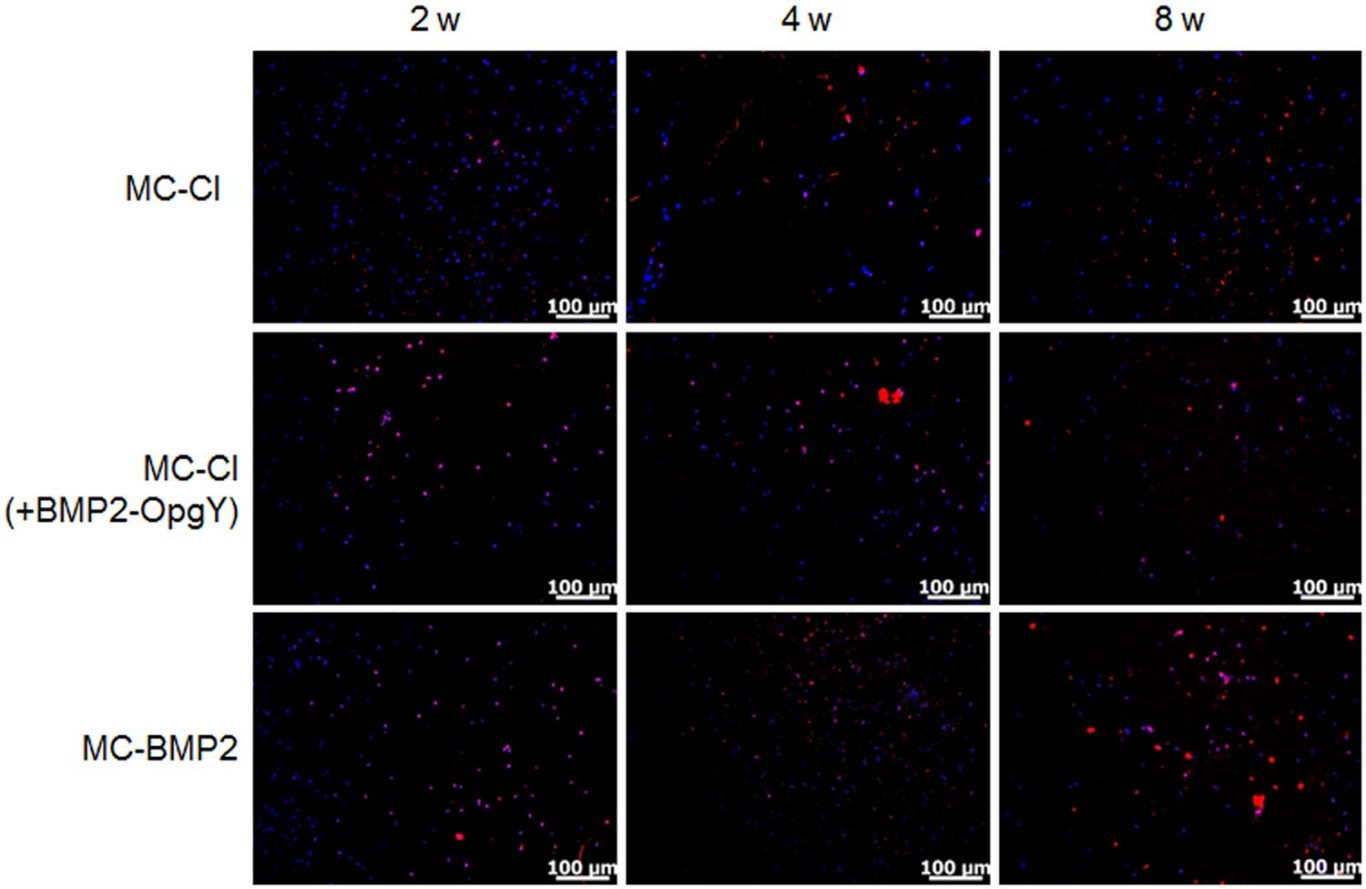
Images of bromodeoxyuridine (BrdU; red) and DAPI (blue) immunofluorescence of MC-Cl, MC-Cl (+BMP2-OpgY), or MC-BMP2 hydrogels removed from mice after 2, 4, or 8 weeks (magnification 200×, scale bars represent 100 μm).

The viability of hPLSCs on the MC-Cl, MC-Cl (+BMP2-OpgY), and MC-BMP2 hydrogels was monitored by fluorescence microscopy of bromodeoxyuridine (BrdU)-pre-labeled hPLSCs (Fig. 6). The BrdU-stained sections of hydrogel implants showed that most hPLSCs (pink) were interspersed throughout the injected hydrogels. These results indicated that BrdU-pre-labeled hPLSCs survived in hydrogel implants through the 8-week implantation period^43, 44^.

**Figure 7:**
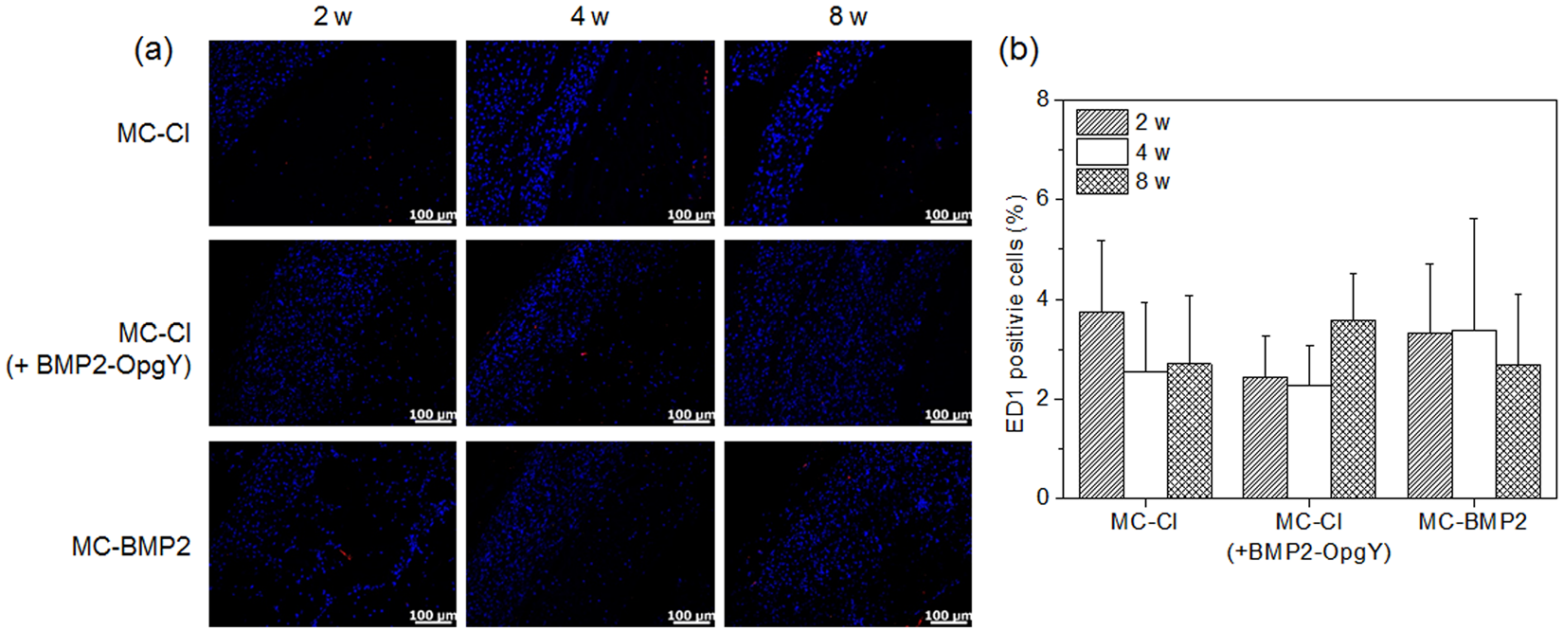
(**a**) Immunofluorescent staining of ED-1 (red) and DAPI (blue) (magnification 200×, scale bars represent 100 μm) (**b**) percentage quantification of the ED-1-positive cells (stained with DAPI) relative to the total number of cells in the section slides removed from nude mice receiving MC-Cl, MC-CL (+BMP2-OpgY), or MC-BMP2 after 2, 4, or 8 weeks.

To assess *in vivo* biocompatibility of the MC-Cl, MC-Cl (+BMP2-OpgY), and MC-BMP2 hydrogels, the tissue sections obtained from the hydrogels were stained with ED1 (a macrophage marker) (Fig. 7a) and DAPI, then were counted to determine the extent of inflammation (Fig. 7b). The hydrogel implants showed ED1 (red) and the host DAPI (blue) staining. The observed ED1 was most likely caused by specific rejection of the MC-Cl, MC-Cl (+BMP2-OpgY), and MC-BMP2 hydrogels. Significantly, lower numbers of ED1-positive cells were observed in all MC-Cl, MC-Cl (+BMP2-OpgY), and MC-BMP2 hydrogels. All hydrogel implants maintained similar numbers of ED1-positive cells from 2 weeks to 8 weeks. Additionally, ED1-positive cells on MC-Cl, MC-Cl (+BMP2-OpgY), and MC-BMP2 hydrogels did not vary significantly. These results indicated the biocompatibility of all MC-Cl, MC-Cl (+BMP2-OpgY), and MC-BMP2 hydrogels.

### Tissue-engineered bone histology

**Figure 8:**
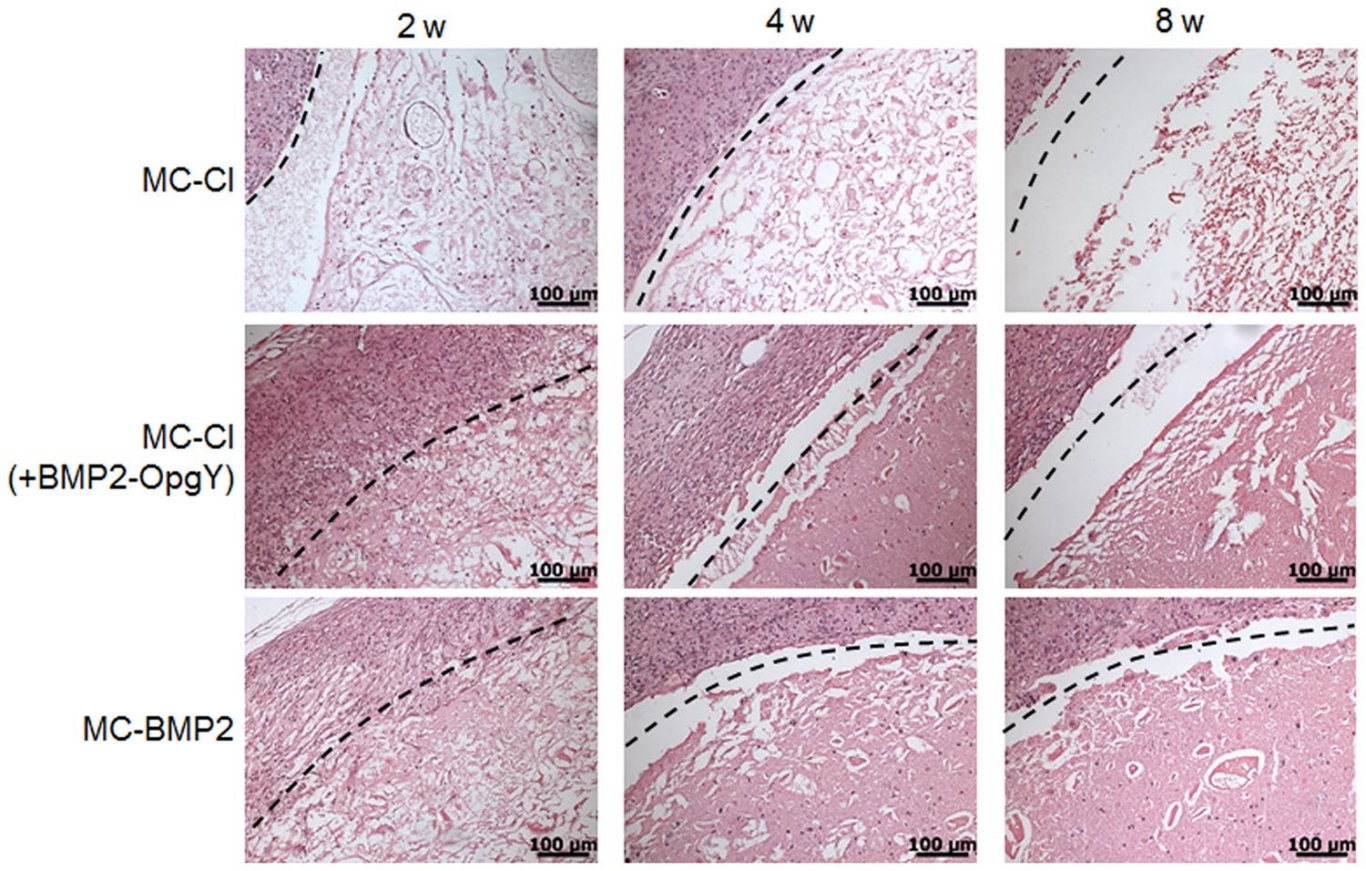
Hematoxylin and eosin-stained histological sections of engineered bone from mice implanted with human periodontal ligament stem cells on MC-Cl, MC-Cl (+BMP2-OpgY), or MC-BMP2 hydrogels removed after 2, 4, or 8 weeks (magnification 200×, scale bars represent 100 μm).

Figure 8 shows H&E-stained histological sections of implants of hPLSC-loaded MC-Cl, MC-Cl (+BMP2-OpgY), and MC-BMP2 hydrogels at 2, 4, or 8 weeks after injection. H&E staining of hydrogel implants allowed them to be clearly distinguished from the underlying host tissue-hydrogel layer. Some host cells and a few new blood vessels were observed within the hydrogels.

**Figure 9:**
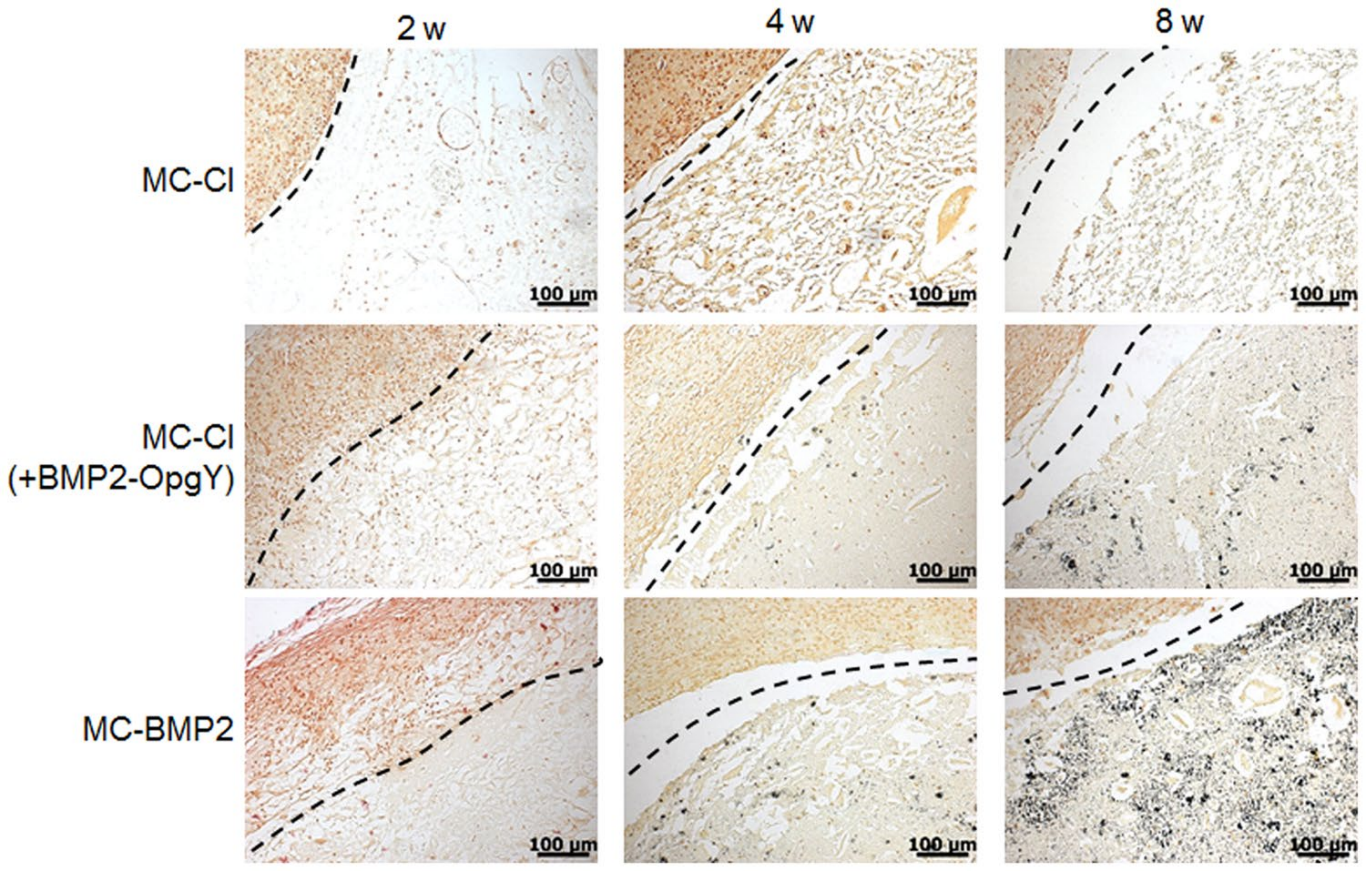
Von Kossa-stained histological sections of engineered bone from mice implanted with human periodontal ligament stem cells on MC-Cl, MC-Cl (+BMP2-OpgY), or MC-BMP2 hydrogels removed after 2, 4, or 8 weeks (magnification 200×, scale bars represent 100 μm).

**Figure 10:**
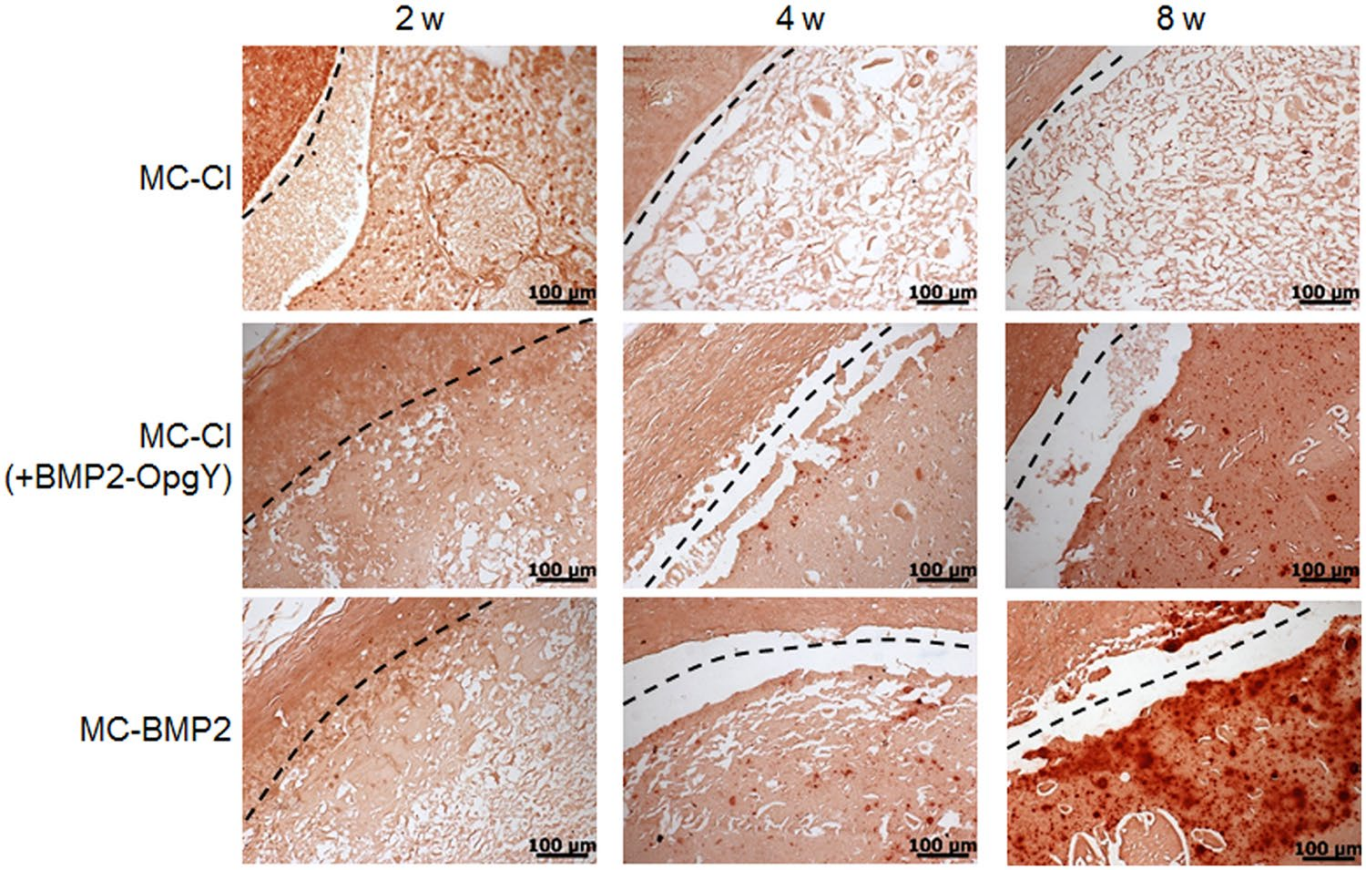
Alizarin Red S-stained histological sections of engineered bone from mice implanted with human periodontal ligament stem cells on MC-Cl, MC-Cl (+BMP2-OpgY), or MC-BMP2 hydrogels removed after 2, 4, or 8 weeks (magnification 200×, scale bars represent 100 μm).

The hydrogel implants taken after 2, 4, or 8 weeks were stained with VK and ARS to monitor the formation of mineralized bone associated with osteogenic differentiation of hPLSCs (Figs 9 and 10). The hPLSC-loaded MC-Cl hydrogel implants showed almost no evidence of mineral deposits within the 8-week implantation period by VK and ARS staining. In the VK- and ARS-stained images of hPLSC-loaded MC-Cl (+BMP2-OpgY), there was a small amount of mineralization only at 8 weeks, indicating that BMP2 slightly induced osteogenic differentiation of hPLSCs.

By contrast, imaged of the VK- and ARS-stained hPLSCs loaded onto MC-BMP2 hydrogels showed some mineralized calcium deposits (VK, black; ARS, red) at 4 weeks post-implantation; these mineral deposits became more evident at 8 weeks after implantation. This result indicated that MC-BMP2 exclusively induced differentiation of hPLSCs toward an osteoblastic phenotype.

### Gene expression in tissue-engineered bone

**Figure 11:**
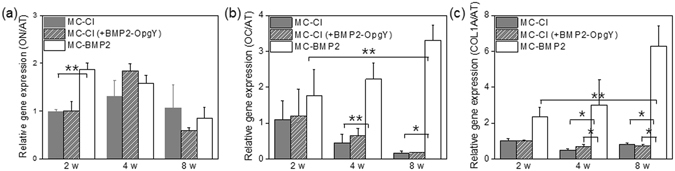
(**a**) Osteonectin (ON), (**b**) osteocalcin (OC), and (**c**) osteopontin (OP) gene expression in human periodontal ligament stem cells on MC-Cl, MC-Cl (+BMP2-OpgY) and MC-BMP2 hydrogels after 2, 4, or 8 weeks. Results were statistically analyzed using a one-way analysis of variance with Bonferroni’s multiple comparison correction (*p < 0.01, **p < 0.05).

The larger proportion of VK- and ARS-staining of MC-BMP2 compared to that of MC-Cl and MC-Cl (+BMP2-OpgY) indicated that osteogenic differentiation occurred in hPLSCs on MC-BMP2. To further characterize the osteogenic differentiation, we performed semi-quantitative real-time PCR to measure the expression of osteonectin (ON), osteocalcin (OC), and collagen type 1α (COL1A) mRNAs (Fig. 11). Alpha tubulin mRNA expression was used as a reference.

As expected, ON served as an early marker of bone matrix synthesis. Expression of ON at 2 weeks in MC-BMP2 hydrogels was about 1.9-fold higher than that in MC-Cl and MC-Cl (+BMP2-OpgY) hydrogels. However, ON expression in MC-Cl (+BMP2-OpgY) was only slightly higher than that in MC-BMP2 at 4 weeks.

OC and COL1A mRNA expression gradually increased in the MC-BMP2 hydrogels as the implantation time increased, whereas OC and COL1A gene expression decreased in the MC-Cl and MC-Cl (+BMP2-OpgY) hydrogels. OC and COL1A mRNA expression in the MC-BMP2 hydrogels was clearly higher than that in the MC-Cl and MC-Cl (+BMP2-OpgY) hydrogels even at week 8.

At 8 weeks, OC and COL1A mRNA expression in the MC-BMP2 hydrogel was about 20-fold and 9-fold higher than that in MC-Cl and MC-Cl (+BMP2-OpgY), respectively. These patterns of expression were consistent with the histology results, which, together, indicate that osteogenic differentiation of hPLSCs was induced by the MC-BMP2 hydrogel.

These results support the hypothesis that MC-BMP2 hydrogels can enhance osteogenic differentiation of hPLSCs better than MC-Cl (+BMP2-OpgY). The covalently immobilized MC-BMP2 employed in this study highly induced osteogenic differentiation of hPLSCs, relative to induction with traditionally loaded BMP2.

In conclusion, we developed the first engineered BMP2-OpgY and an injectable MC-BMP2 formulation for osteogenic differentiation of hPLSCs. Specifically, covalently immobilized MC-BMP2 induced synergistic osteogenic differentiation on hPLSCs better than a physically BMP2-loaded MC hydrogel. We anticipate that the injectable *in situ*-forming MC-BMP2 hydrogel investigated here may be used for noninvasive administration of therapies for debilitating orthopedic conditions.

## Methods

### Materials

MPEG (number-average molecular weight *M*_n_ ≈ 750, Sigma; MO, USA) and HCl (1.0 M solution in diethyl ether, Sigma; MO, USA) were used as received. ε-Caprolactone (CL) (TCI; Tokyo, Japan) was distilled over CaH_2_ in reduced pressure. 2-Chlorocyclohexanone (TCI; Tokyo, Japan), 3-chloroperoxybenzoic acid (Sigma; MO, USA), and sodium azide (Sigma; MO, USA) were used as received. CH_2_Cl_2_ was distilled sequentially from CaCl_2_ and CaH_2_ under nitrogen before use.

### Characterization

^1^H nuclear magnetic resonance (NMR) spectra were measured using a Mercury Plus 400 (Varian) with CDCl_3_ in the presence of tetramethylsilane as an internal standard. The molecular weight distributions of MC-Cl, MC-N_3_, and MC-NH_2_ were measured at 40 °C using a YL-Clarity GPC system (YL 9170 RI detector) equipped with three columns (Shodex K-802, K-803, and K-804 polystyrene gel columns). For this measurement, CHCl_3_ was used as the eluent at a flow rate of 1.0 mL/min, with polystyrene used for calibration.

### Construction of BMP2 expression plasmids

The synthesized mature human BMP2 gene with an *N*-terminal His_6_ tag (DNA sequence ATGGGCAGCTATGGTAGTCATCACCATCATCACCACGGATCCCAGGCGAAACACAAACAGCGTAAACGTCTGAAATCTTCTTGCAAACGTCACCCGCTGTACGTTGACTTCTCTGACGTTGGTTGGAACGACTGGATCGTTGCGCCACCGGGTTACCATGCGTTCTACTGCCACGGTGAATGCCCGTTCCCGCTGGCGGACCACCTGAACTCTACCAACCACGCGATCGTTCAGACCCTGGTTAACTCCGTTAATTCTAAAATCCCGAAAGCGTGCTGCGTTCCGACCGAACTGTCTGCGATCTCTATGCTGTACCTGGACGAAAACGAAAAAGTTGTTCTGAAAAACTACCAGGACATGGTTGTTGAAGGTTGCGGTTGCCGTTAA) was cloned into pET28a (Novagen) using the NcoI and HindIII sites (pSPEL112). To construct a plasmid for the expression of BMP2 with *O*-propargyl tyrosine (OpgY) (BMP2-OpgY), an amber codon (TAG) was introduced at the fourth position in place of Tyr via polymerase chain reaction (PCR) using a pair of primers (5′-ATATACCATGGGCAGCTAGGGTAGTCATC-3′ and 5′-AATTCCAAGCTTTTAACGGCAACCGCAACCTTCAACAACCATG-3′; the NcoI and HindIII, respectively, are underlined) (pSEPL117). The amino acid sequence of BMP2 was as follows: MGSYGSHHHHHHGSQAKHKQRKRLKSSCKRHPLYVDFSDVGWNDWIVAPPGYHAFYCHGECPFPLADHLNSTNHAIVQTLVNSVNSKIPKACCVPTELSAISMLYLDENEKVVLKNYQDMVVEGCGCR. The amino acid sequence of BMP2-OpgY was as follows: MGS*GSHHHHHHGSQAKHKQRKRLKSSCKRHPLYVDFSDVGWNDWIVAPPGYHAFYCHGECPFPLADHLNSTNHAIVQTLVNSVNSKIPKACCVPTELSAISMLYLDENEKVVLKNYQDMVVEGCGCR (* = OpgY).

### Expression of BMP2 and BMP2-OpgY

To express wild-type BMP2, pSPEL112 was transformed into *E. coli* BL21 (DE3). The cells were cultured in 2× YT medium at 37 °C until the OD_600_ reached ~0.5, and then 1 mM isopropyl β-D-1-thiogalactopyranoside was added to induce expression of BMP2. In the case of BMP2-OpgY, the plasmid for BMP2-OpgY expression (pSPEL117) and the plasmid encoding an orthogonal pair of aminoacyl-tRNA synthetase and tRNA for incorporation of the amino acid analogue into the amber codon (pEVOL) were transformed into *E. coli* BL21(DE3)^45, 46^. We used a pEVOL plasmid encoding aminoacyl-tRNA synthetase to incorporate p-azido-l-phenylalanine (AzF) into the amber codon, because the enzyme was reported to be active toward OpgY as well as AzF^46^. The cells were cultured in 2xYT medium at 37 °C until the OD_600_ reached ~0.5, and then 0.2 % L-arabinose, 1 mM isopropyl β-D-1-thiogalactopyranoside and 1 mM OpgY were added to the culture to induce expression of aminoacyl-tRNA synthetase and BMP2-OpgY. After 3 h, the cells were harvested by centrifugation (9,000 × g at 4 °C for 15 min) and stored at −20 °C until they were used.

### Purification of BMP2 and BMP2-OpgY

BMP2 and BMP2-OpgY were purified following methods reported by Hillger *et al*. with some modifications^47^. BMP2 formed inclusion bodies under the expression conditions, and the insoluble fraction was isolated. The frozen cell pellet was thawed in TBST (50 mM Tris-HCl (pH 7.5), 150 mM NaCl, and 1% Triton X-100), and then cell membranes were disrupted by sonication. The cellular lysate was centrifuged at 6,000 × *g* and 4 °C for 15 min, and then the insoluble fraction was washed three times with TBST. Following this, the washed pellet was solubilized with lysis buffer (6 M guanidium-HCl, 20 mM Tris-HCl (pH 8.0), and 14 mM 2-mercaptoethanol) at 37 °C for 1 h. The solution was centrifuged at 15,000 × *g* and 25 °C for 15 min, and then the supernatant containing BMP2 was diluted in refolding buffer (0.1 M Tris-HCl [pH 8.0], 1 M arginine, 2 mM reduced glutathione, and 5 mM EDTA) at a 1:100 ratio and refolded by stirring at 4 °C for 14 days. Biologically active BMP2 exits as a dimer, and we purified the dimer form using a heparin column (HiTrap Heparin HP, GE Healthcare). Urea pellets were added to the BMP2 solution to reach a concentration of 6 M, and the pH of the solution was adjusted to 5.5 by adding acetic acid. The protein solution was loaded into a heparin column equilibrated with Buffer A (0.1 M Tris-acetate [pH 5.5], 6 M urea, and 5 mM EDTA), and dimeric BMP2 was eluted with an NaCl gradient from 0 M to 1 M in Buffer A. The fractions containing dimeric BMP2 were pooled and dialyzed against 10 mM HCl. Then the solution was lyophilized.

### hPLSC culture and characterization

Fresh hPLSCs were isolated from third molar teeth that had been extracted from healthy, non-smoking young woman (aged 22 years) at Inha Hospital, Korea. The research adhered to the tenets of the Declaration of Helsinki and informed consent was obtained from woman patient. All procedures for this study were approved by the Institutional Review Board for the Human Subjects Research and Ethics Committee of Inha Hospital (approval no. IUH IRB 12-150). hPLSCs were cultured in a 75-cm^2^ tissue culture flask (BD Falcon; CA, USA) in α-minimal essential medium (α-MEM; Gibco; NY, USA) containing 15% fetal bovine serum (FBS; Gibco; NY, USA), 2 mmol/L l-glutamine (Gibco; NY, USA), 100 μmol/L ascorbic-acid-2-phosphate (Sigma; MO, USA), and 1% penicillin-streptomycin (PS; Gibco; NY, USA) in 5% CO_2_ at 37 °C. Fifth-passage hPLSCs were used for this experiment.

hPLSCs were identified by flow cytometry using a FACScan cytometer (BD Bioscience; CA, USA). CD90 and CD166 cell surface antigens were considered positive markers, whereas CD34 were considered a negative marker. The hPLSCs expressed mesenchymal stem cell markers CD90 (>99.9%) and CD166 (99.8%), but showed no expression of the hematopoietic stem cell marker CD34 (<0.01%).

For the BrdU-tagging experiment, hPLSCs were labeled with BrdU by incubation for 24 h at 37 °C using BrdU (Sigma; MO, USA).

### *In vitro* osteogenic differentiation of hPLSCs

Passage five hPLSCs (1 × 10^4^) were seeded into 6-well plates (Nunc; Roskilde, Denmark) and incubated for 24 h. The seeded hPLSCs were cultured for 4 weeks with culture medium as described in the previous section (control). For osteogenic induction, the seeded hPLSCs were then treated with the osteogenic medium α-MEM supplemented with 15% FBS and 1 μg/mL BMP2 (naïve BMP2 [Cellumed, Seoul, Korea] or BMP2-OpgY). For the single BMP2-OpgY treatment, the hPLSCs were treated with osteogenic medium, which was replaced by culture medium after 3 days and cultured for an additional 4 weeks. For the repeated naïve BMP2 or BMP2-OpgY treatment, the hPLSCs were treated with osteogenic medium that was exchanged for fresh osteogenic medium with naïve BMP2 or BMP2-OpgY every 3 days for 4 weeks. To confirm osteogenic induction of hPLSCs, tissues were stained with ALP, ARS and VK after 1, 2, 3, or 4 weeks of culture.

For ALP staining, the cells were stained using a Leukocyte Alkaline Phosphatase Kit (Sigma; MO, USA) based on naphthol AS-BI, following the manufacturer’s protocol. For ARS staining, the cells were washed with phosphate buffered saline (PBS) and fixed in 4% paraformaldehyde (Biosesang Inc.; Gyeonggi, Korea). After washing three times with deionized water, 0.2% ARS solution was incubated with the fixed cells for 15 min. For VK staining, the cells were washed twice with PBS and then fixed using 4% paraformaldehyde (Biosesang Inc.; Gyeonggi, Korea). The fixed cells were incubated in 5 wt% silver nitrate (Sigma; MO, USA) solution for 1 h and then washed with deionized water. The color staining was developed with exposure to sodium carbonate/formalin solution for 1 min. The nuclei of cells were stained with Nuclear Fast Red (Sigma; MO, USA). All experiments were performed at least 3 times.

### Preparation of 2-chloro-ε-caprolactone (fCL)

All glasses were heated in a vacuum and flushed with a dry nitrogen stream. All reactions were carried out under a dry nitrogen stream. 3-Chloroperoxybenzoic acid (3.38 g, 20 mmol) was placed in a 100 mL flask and dissolved in CH_2_Cl_2_ (39 mL). 2-Chlorocyclohexanone (2 g, 15 mmol) was added to the solution, which was held at room temperature. After 48 h, the resulting solution was washed with a NaHSO_3_-saturated aqueous solution three times and a NaHCO_3_ aqueous solution several times, followed by washing with brine solution. The organic phase was dried over anhydrous magnesium sulfate and concentrated by evaporation to yield fCL in a light yellowish viscous liquid. The obtained mixture was purified by silica gel column chromatography using a solution of *n*-hexane and ethylacetate (v/v = 70/30, Rf = 0.6) as an eluent to yield fCL in a clear liquid. ^1^H NMR (CDCl_3_): 4.81 (*t*, 1 H, -CH(Cl)-) 4.61 (*t*, 1 H, -OCH_2_-), 4.22 (*t*, 1 H, -OCH_2_-), 2.05 (*m*, 4 H, -CH_2_-, -CH_2_-), 1.82 (*m*, -CH_2_-).

### Synthesis of the MC-Cl

All glasses were heated in a vacuum and flushed with a dry nitrogen stream. The typical polymerization process to produce MC-Cl with a CL and fCL ratio of 91:9 using MPEG (750 g/mol) as an initiator was as follows: MPEG (0.94 g, 1.25 mmol) and toluene (30 mL) were added to a flask. Azeotropic distillation was performed to remove water from the MPEG and toluene. Under a dry nitrogen stream, toluene was distilled. CL (2.62 g, 23.0 mmol) and fCL (0.38 g, 2.6 mmol) were introduced into the MPEG at room temperature, followed by the addition of a 1.0 M solution of HCl in diethyl ether (1.7 mL, 1.7 mmol) at 25 °C. After 24 h, the mixture was poured into *n*-hexane to precipitate polymers. Precipitated polymers were obtained from the supernatant by decantation, dissolved in CH_2_Cl_2_, and then filtered. The resulting polymer solution was concentrated by rotary evaporation and dried in a vacuum to yield a colorless polymer of 90% yield.

### Synthesis of the MC-N_3_

MC-Cl (3 g, 0.97 mmol) and DMF (30 mL) were introduced into a flask. Sodium azide (0.25 g, 3.9 mmol) was added to the MC-Cl solution at room temperature under nitrogen, and the mixture was stirred at 70 °C for 24 h. The reaction mixture was then poured into *n*-hexane to precipitate the polymer, which was separated from the supernatant by decantation. The obtained polymer was redissolved in CH_2_Cl_2_. The resulting solution was washed with brine solution. The organic phase was concentrated using a rotary evaporator and dried in a vacuum to obtain a yellowish polymer of 89% yield. An element analysis resulted in calculated values for MC-N_3_: C, 59.9; H, 8.6; and N, 3.6; and measured values of C, 60.1; H, 9.2; and N, 3.2.

### Preparation of the MC-BMP2

MC-N_3_ (1 g, 0.325 mmol,) 0.4 mM CuSO_4_, 20 mM ascorbate, and 4 mL of PBS were introduced into a flask. BMP2-OpgY (100 μg) was added to the MC-N_3_ solution at room temperature under nitrogen, and the mixture was stirred at room temperature. After 3 h, the reaction mixture was poured into a dialysis tube (molecular weight cut-off: 3.5 kDa, Spectrum Laboratories, CA, USA) and dialyzed for 3 days to remove unreacted CuSO_4_ and ascorbate. The dialyzed solution was cooled to −80 °C and freeze-dried to yield the final MC-BMP2 of 90% yield.

### Characterization of MC-BMP2

The obtained MC-BMP2 was solubilized in 50 μL of distilled water (DW). One microliter of NuPAGE LDS buffer (4×) (Invitrogen; CA, USA) and 2.5 μL of NuPAGE reducing agent (10×) (Invitrogen; CA, USA) were added to 6.5 μL of MC-BMP2 solution. For the control, 1 μL of NuPAGE LDS buffer and 2.5 μL of NuPAGE with 500 mM dithiothreitol as reducing agent were added to 2 μL of native BMP2-OpgY (1 μg/μL) solution. Native BMP2-OpgY and MC-BMP2 were individually incubated at 70 °C for 15 min and then loaded onto a NuPAGE 4–12% Bis-Tris gel (Invitrogen; CA, USA), followed by electrophoresis at 200 mV using XCell SureLock (Invitrogen; CA, USA). MC-BMP2 and native BMP2 were visualized by staining with 0.025% Coomassie Blue.

### Viscosity measurements

To determine the gelation time, the prepared MC-Cl, MC-Cl (+BMP2-OpgY), and MC-BMP2 solutions were incubated overnight at 4 °C. The viscosities of the copolymer solutions were measured using a Brookfield Viscometer DV-III Ultra in a vessel with a tight cap to prevent evaporation of water, which was equipped with a programmable rheometer and circulating baths with a programmable TC-502P controller (Brookfield Engineering Laboratories, Middleboro, MA). The viscosities of the copolymer solutions were determined using a T-F spindle at 0.1 rpm and temperatures between 10 °C and 52 °C (raised in increments of 1 °C/min).

### *In vitro* release of BMP2-OpgY

100 µL of MC-Cl (+naïve BMP2) (20 µg/mL naïve BMP2) and MC-BMP2 (20 µg/mL BMP2-OpgY) hydrogels were individually loaded onto each 24-transwell membranes (Corning, Lowell, MA, USA). Bottom of 24-transwell plate was filled with 1 mL of PBS, and shake at 100 rpm in an incubator at 37 °C. The PBS was removed from each well and replaced with same volume of fresh PBS. The amount of naïve BMP-2 or BMP2-OpgY in the PBS was measured using an ELISA kit (BMP-2 Quantikine ELISA Kit; R&D systems, MN, USA). The solution absorbance was measured at 450 nm using a plate-reader (EL808 Ultra Microplate Reader, Bio Tek Instruments, USA). The concentration of standard solution of naïve BMP-2 or BMP2-OpgY was measured using an ELISA kit and the amount of cumulatively released naïve BMP-2 or BMP2-OpgY was calculated by comparison with each standard calibration curve prepared through measurements of solution of naïve BMP-2 or BMP2-OpgY in PBS with various known concentrations. All experiments were performed three times.

### Cytotoxicity tests on hydrogel

A solution of MC-Cl, MC-Cl (+naïve BMP2), MC-Cl (+BMP2OpgY), and MC-BMP2 at RT was mixed with hPLSCs (1 × 10^6^ cells). Then hPLSCs-loaded hydrogels (100 uL) was separately plated onto the wells of 24-transwell membranes. For the control group, hPLSCs alone in equivalent numbers were seeded into the 24-transwell membranes. Cytotoxicity of hPLSCs was examined at 1, 4 and 7 days at 37 °C in a humidified incubator. The cytotoxicity was measured using a WST-1 Kit (Roche, Mannheim, Germany). WST-1 reagent (100 μL) was added to the wells and the plates were incubated at 37 °C for 4 h. The samples were then gently mixed, and an aliquot (100 μL) from each well was transferred to a 96-well plate (SPL Lifescience, Gyeonggi-do, Korea). The absorbance of the solution at 450 nm was measured using a microplate reader (EL808 Ultra Microplate Reader, Bio Tek Instruments, USA). The experiments of cytotoxicity were performed three times.

### *In vivo* implantation

The protocol of animal experiments was approved by the Institutional Animal Experiment Committee (Approval No. 2016-0018) at Ajou University School of Medicine and all animals were treated in accordance with the guidelines for the Care and Use of Animals for experimental and Scientific purposes. Thirty-six male nude mice (20–22 g, 6 weeks old), divided into three groups of 12 mice each, were used in animal tests for 2, 4, or 8 weeks. The three experimental groups of MC-Cl (+hPLSCs), MC-Cl (+BMP2-OpgY, +hPLSCs), and MC-BMP2, +hPLSCs) were prepared by gently mixing solutions of MC-Cl, MC-Cl (+BMP2-OpgY), and MC-BMP2 with 5 × 10^6^ BrdU-labeled hPLSCs. Two formulation types (MC-Cl [+BMP2-OpgY] and MC-BMP2) containing the same amount of BMP2-OpgY (20 μg) were used for *in vivo* implantation. Within 5 min of mixing, a 200-μL syringe with a 26-gauge needle was used to inject the solution into the subcutaneous dorsum of a nude mouse that had been anesthetized with 200 μL/kg of Zoletil-Rompun (1:1 solution). The resulting MC-Cl, MC-Cl (+BMP2-OpgY), and MC-BMP2 hydrogel implants were then allowed to develop and were biopsied at 2, 4, or 8 weeks.

### FE-SEM measurements

The morphologies of the MC-Cl, MC-Cl (+BMP2-OpgY), and MC-BMP2 hydrogels formed *in vivo* was examined by FE-SEM (LEO Supra 55; Carl Zeiss; Oberkochen, Germany). The hydrogels were excised at 2, 4, or 8 weeks after implantation and immediately mounted on a metal stub pre-cooled with liquid nitrogen. The stub was coated with a thin layer of gold in an argon atmosphere using a plasma-sputtering apparatus (PS-1200; Paraone; Seoul, Korea) and then examined by FE-SEM.

### Histological analysis

Mice were sacrificed at 2, 4, or 8 weeks after implantation, and the hydrogel implants were individually dissected and removed from the subcutaneous dorsum. The tissues were immediately fixed with 10% formalin and embedded in paraffin. The embedded specimens were sectioned (4 µm) along the longitudinal axis of the implant. The sectioned slides were incubated at 70 °C for 2 h to deparaffinize and hydrated with a series of 100, 95, 80, 70, and 60% ethyl alcohol. Antigen was retrieved using a citrate buffer solution at 120–130 °C for 10 min. The slides were washed with Tris buffered saline (TBS) for 5 min and then blocked with 5% bovine serum albumin (BSA; Roche, Penzberg, Germany) in PBS for 30 h at 37 °C. The sections were incubated for 16 h at 4 °C with anti-BrdU antibodies in 1% BSA in TBS (1:650). The slides were washed three times with TBS and then incubated with secondary antibody (goat anti-mouse Alexa Fluora 594; Invitrogen, CA, USA) in 1% BSA in TBS (1:200) for 30 min at room temperature in the dark. The slides were washed again with TBS, counterstained with DAPI, and then mounted using fluorescent mounting solution (DAKO; Glostrup, Denmark).

For hematoxylin and eosin (H&E) staining, the paraffinized slides were deparaffinized with xylene and hydrated using a series of 100, 95, 80, 70, and 60% ethyl alcohol. Then, slides were washed in running tap water and stained with hematoxylin for 3 min and then washed with DW. The slides were then stained with eosin for 2 min and washed with DW. Thereafter, stained slides were hydrated using 95 and 100% ethyl alcohol and xylene for 2 min, and then fixed and mounted with mounting medium (Muto Pure Chemicals; Tokyo, Japan).

For VK staining, the slides were washed with DW and treated with 5 wt% silver nitrate (Sigma; MO, USA) solution for 1 h, and then washed three times with DW. The color staining was developed with exposure to a sodium carbonate/formalin solution for 1 min. The nuclei of cells in tissues were stained with Nuclear Fast Red (Sigma; MO, USA).

For ARS staining, the slides were washed with DW and treated with Alizarin Red S solution (Sigma; MO, USA) solution for 30 min, and then washed with acetone and hydrated using xylene. The stained tissues were fixed and mounted with mounting medium (Muto Pure Chemicals; Tokyo, Japan).

For macrophages (ED1) staining, the slides were incubated for 10 min at 120–130 °C in citrate buffer solution (Sigma; MO, USA) and then incubated for 10 min in PBS and washed with PBS contained with 0.05% Tween-80 (PBST, Sigma, St. Louis, MO, USA) in PBS. The slides were blocked in PBS containing horse serum (HS; Gibco, Auckland, New Zealand) and 5% bovine serum albumin (BSA; Roche, Penzberg, Germany) for 90 min at 37 °C. The slides were incubated at 4 °C for 16 h with ED1 (mouse anti-rat CD68; Serotec, Oxford, UK) in antibody diluent (DAKO; Glostrup, Denmark) (1:1000) and incubated for 12–16 h at 4 °C and then with PBS and PBST. The slides were incubated with a secondary antibody (goat anti-mouse Alexa Fluora 594; Invitrogen; CA, USA) (1:200) and incubated for 3 h. The slides were washed again with PBST, counterstained with DAPI, and mounted with a fluorescent mounting solution (DAKO; Glostrup, Denmark).

All slides were visualized under an LSM 710 microscope (Carl Zeiss Microimaging GmbH; Göttingen, Germany) and analyzed with ZEN 2009 software (Carl Zeiss Microimaging GmbH; Göttingen, Germany).

### RNA isolation and reverse transcription-PCR (RT-PCR)

RNA was isolated from the excised hydrogels with TRIzol solution (Invitrogen; CA, USA). RNA was quantified using an ND-1000 spectrophotometer (NanoDrop Technologies; DE, USA) at 260 nm, and the concentration and quality of RNA were confirmed by agarose gel electrophoresis. RNA samples (50 ng) were then reverse transcribed using a SuperScript III First-Strand Kit (Invitrogen).

PCR amplification and real-time fluorescence detection of osteonectin (ON), osteocalcin (OC), and collagen type 1 α (COL1) were performed with a Chromo4 Real-Time PCR System (Bio-Rad; CA, USA), and Power SYBR Green (Applied Biosystems; UK).

Expression of the ON, OC, and COL1 genes was normalized to that of alpha tubulin (AT). All samples were analyzed in triplicate. Data analysis was conducted using the comparative cycle threshold (Ct) method (2^−ΔΔCt^) for relative quantification. The PCR primers (GenoTech; Daejeon, Korea) used were as follows:

ON, 5′-ATCTTCCCTGTACACTGGCAGTTC-3′, 5′-CTCGGTGTGGGAGAGGTACC-3′; OC,5′-CCTATTGGCCCTGGCCGCAC-3′, 5′-ACTGGGGCTCCCAGCCATTGA-3′; COL1, 5′-CCTGGATGCCATCAAAGTCT-3′, 5′-TCTTGTCCTTGGGGTTCTTG-3′ and AT, 5′-TGGAACCCACAGTCATTGATGA-3′, and 5′-TGATCTCCTTGCCAATGGTGTA-3′.

### Statistical analysis

All the gene expression values were obtained from multiple independent experiments. All data are presented as the mean and standard deviation (SD). The results were analyzed by one-way analysis of variance (ANOVA) using SPSS 12.0 software (SPSS Inc.; IL, USA).

## Electronic supplementary material

Supplementary information
