# Supplementary material for: BMP2-modified injectable hydrogel for osteogenic differentiation of human periodontal ligament stem cells
Source: Sci Rep. 2017 Jul 26;7:6603. doi: 10.1038/s41598-017-06911-8 (PMC5529463; doi:10.1038/s41598-017-06911-8)
Supplement: Supplementary file 1 — Supplementary information [file 41598_2017_6911_MOESM1_ESM.doc]

**Supplementary Information**

**BMP2-immobilized injectable hydrogel for osteogenic differentiation of human periodontal ligament stem cells**

Seung Hun Park, Jin Seon Kwon, Byeong Sung Lee, Ji Hoon Park, Bo Keun Lee, Jeong-Ho Yun, Bun Yeoul Lee, Jae Ho Kim, Byoung Hyun Min, Tae Hyeon Yoo*, Moon Suk Kim*


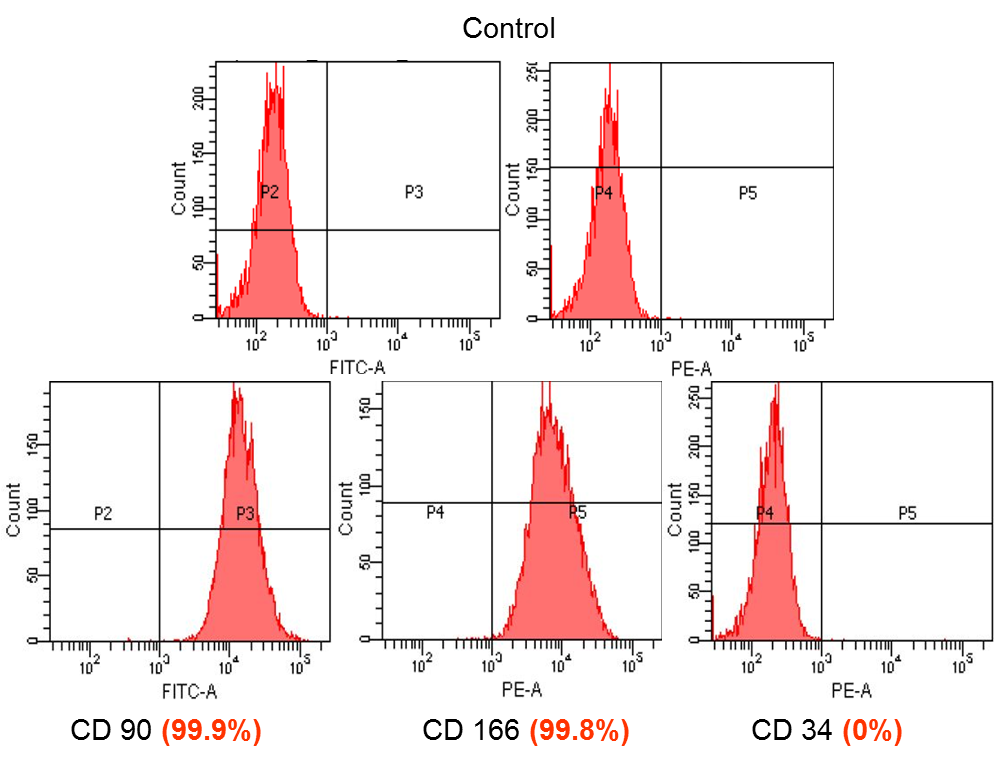


**Supplementary Figure S1.** The expression of surface antigens by passage five hPLSCs.


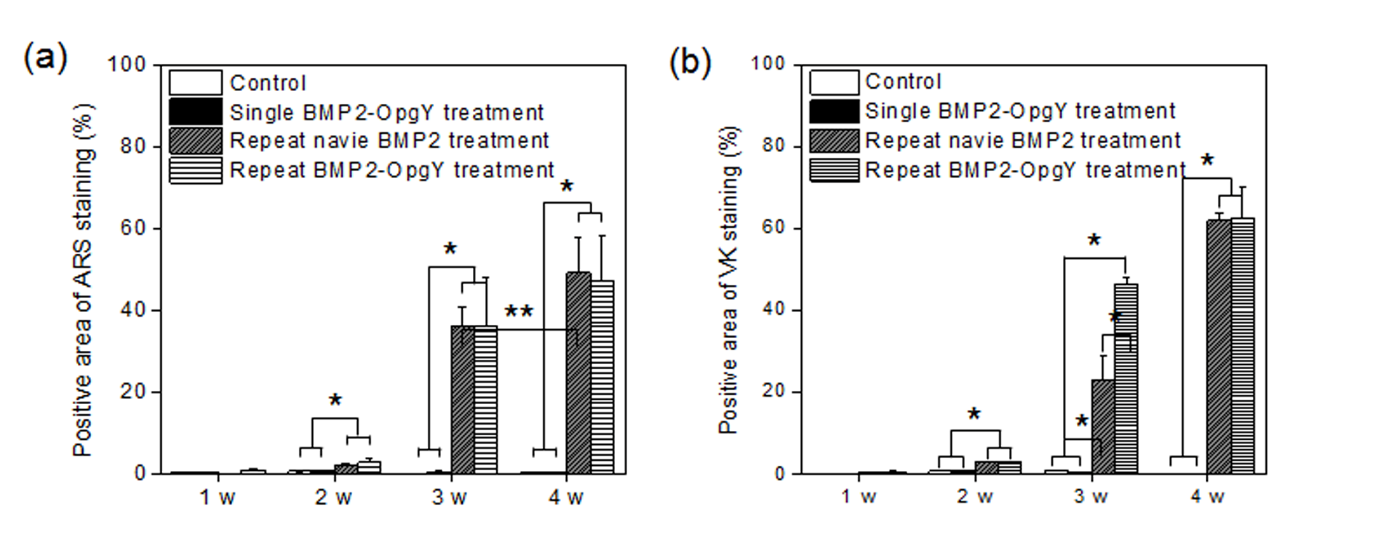


**Supplementary Figure S2.** Quantitative analysis of (a) alizarin red S and (b) von Kossa staining for *in vitro* osteogenic differentiation by naïve BMP2 or BMP2-OpgY. (**p* < 0.001, ***p* < 0.05)


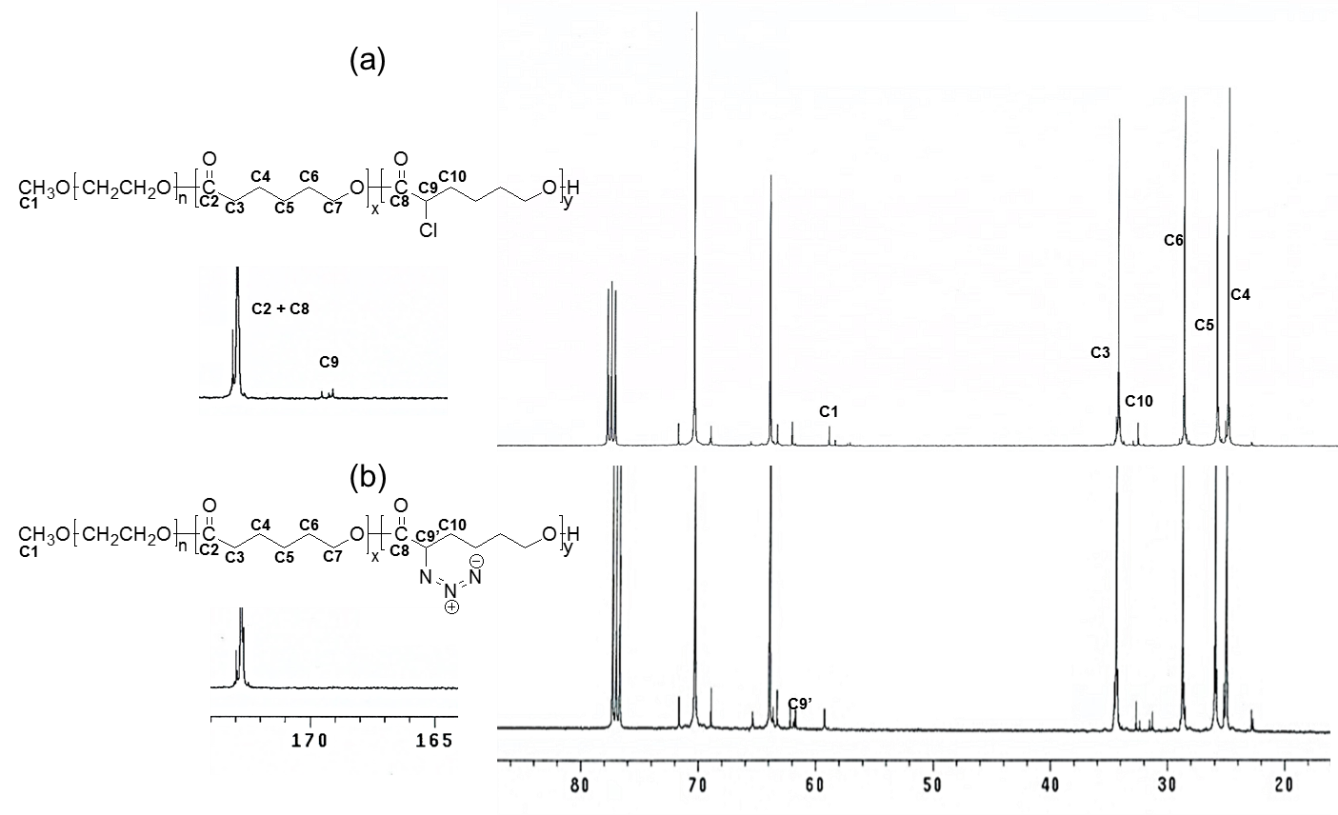


**Supplementary Figure S3.** ^13^C-NMR spectra of (a) MC-Cl and (b) MC-N_3_.

**Supplementary Figure S4**. *In vitro* percentage cumulative release profile of naïve BMP-2 or BMP2-OpgY from MC-Cl (+naïve BMP2) or MC-BMP2 at 37^o^C in PBS.


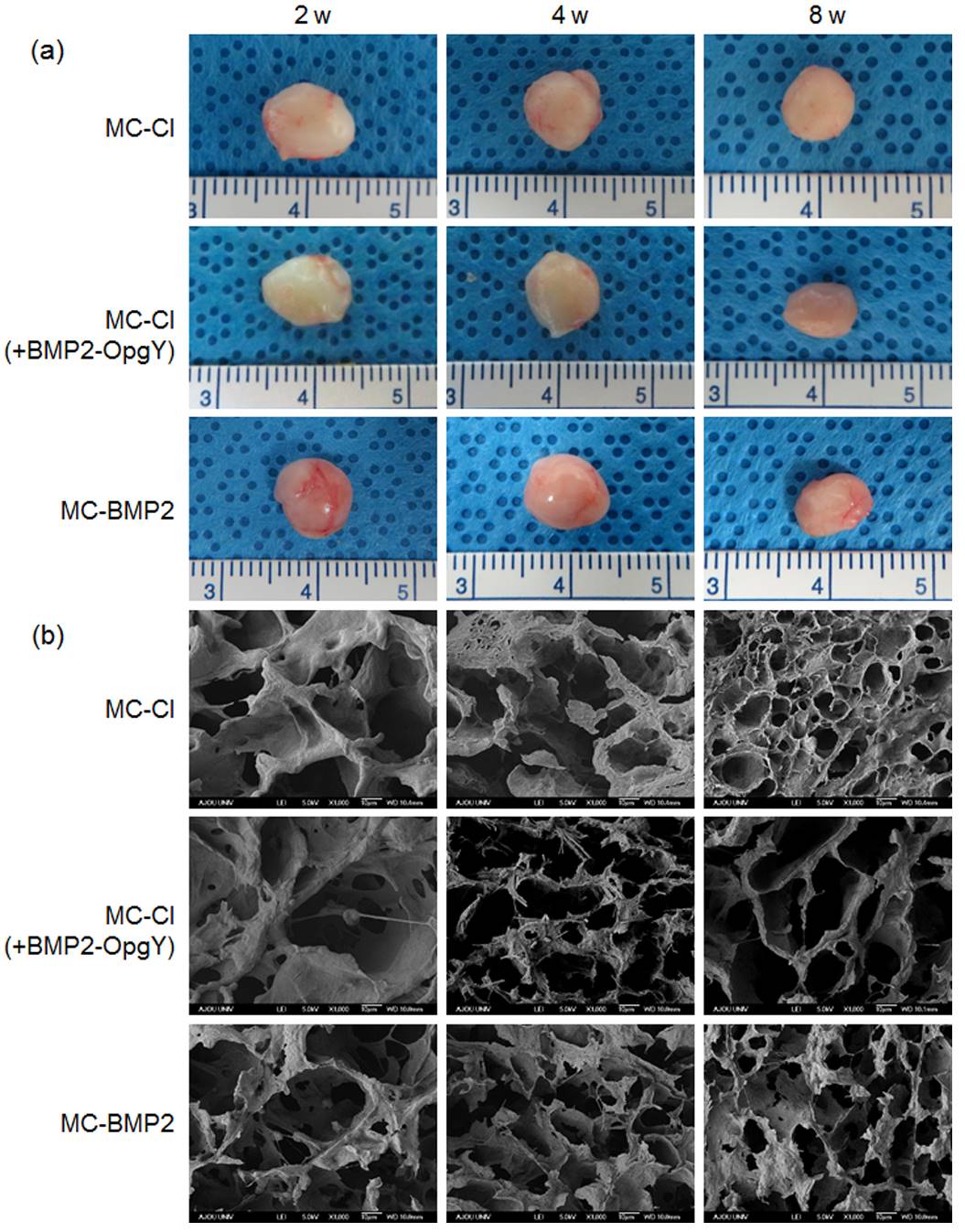


Supplementary Figure S5. (a) Optical images and (b) scanning electron micrographs of MC-Cl, MC-Cl (+BMP2-OpgY), or MC-BMP2 hydrogels removed from mice after 2, 4, or 8 weeks (magnification 1000×).


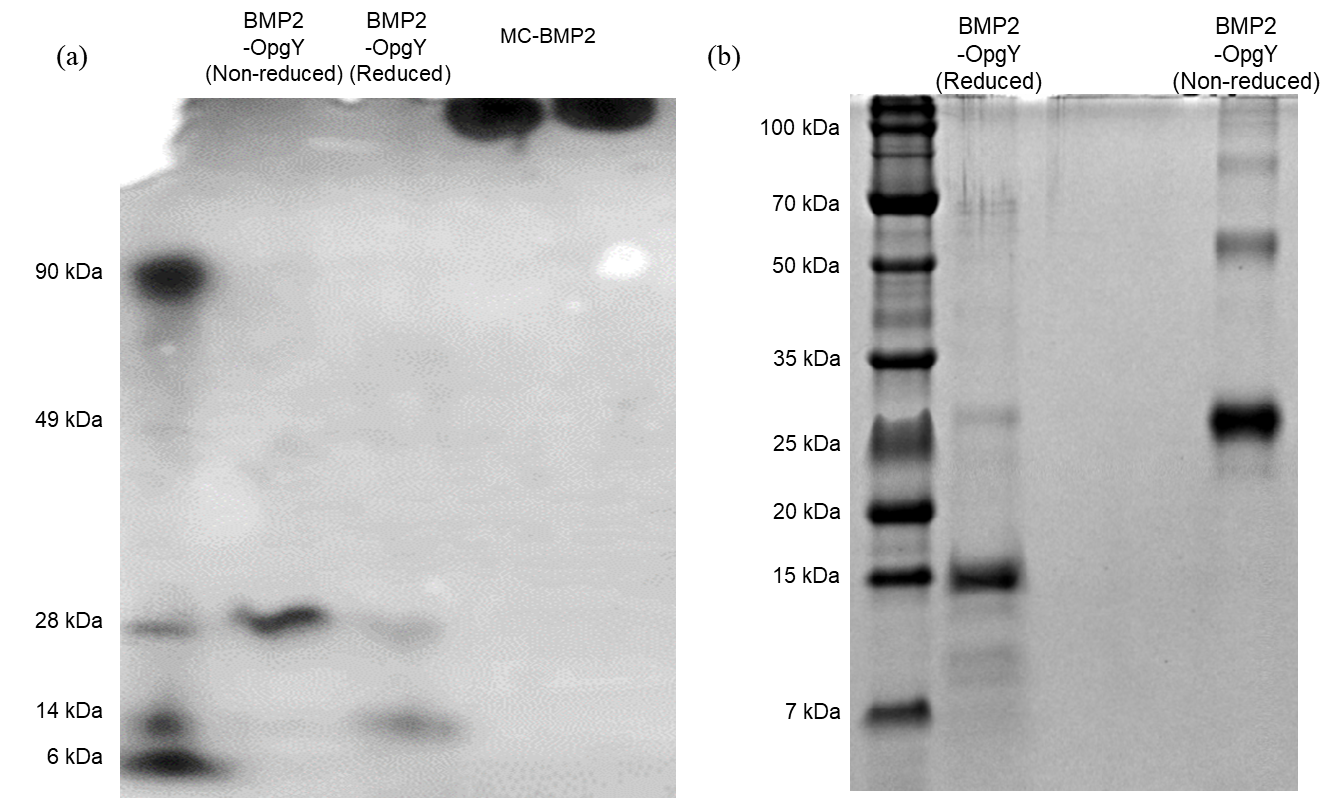


Supplementary Figure S6. (a) The uncropped full-length gel of Fig. 1(c). (b) The uncropped full-length gel of Fig. 2(d).
